# Not All Bone Lesions After Breast Cancer Treatment Indicate Metastasis: A Case Report and Review of the Literature

**DOI:** 10.7759/cureus.96191

**Published:** 2025-11-06

**Authors:** Mohamed Kaakoua, Aznag Mohamed Amine, Nada Ebbadi, Soukayna Boujmadi, Moussa Abdolaziz Sawadogo, Hamza Laabbar, Yahyaoui Hicham, Ismail Essadi

**Affiliations:** 1 Medical Oncology, Ibn Sina Military Teaching Hospital, Marrakesh, MAR; 2 Clinical Hematology, Ibn Sina Military Teaching Hospital, Marrakesh, MAR; 3 Radiology, Ibn Sina Military Teaching Hospital, Marrakesh, MAR; 4 Hematology and Immuno-Hematology, Ibn Sina Military Teaching Hospital, Marrakesh, MAR

**Keywords:** bone metastasis, breast cancer, case report, diagnosis, lytic bone lesion, multiple myeloma

## Abstract

Breast cancer is one of the osteophilic cancers. However, the appearance of bone-related symptoms in a pretreated patient is not always indicative of bone metastases. We report a case of a patient pretreated for localized breast cancer who subsequently developed bone symptoms, with lytic bone lesions on spinal MRI, which were ultimately found to be related to multiple myeloma. This clinical case outlines the diagnostic difficulties of multiple myeloma in patients with a history of breast cancer, highlighting the risk of delayed multiple myeloma management.

## Introduction

Breast cancer is the most common cancer among women in Morocco. Its incidence is about 45.5 cases per 100,000 inhabitants [1]. Bone is the most favored site for breast cancer metastasis [2]. However, the appearance of bone manifestations in a patient treated for localized breast cancer is not always related to a bone relapse of this cancer. We describe through this clinical case the diagnostic difficulties of multiple myeloma in a patient with a history of breast cancer and who is at risk of delaying the management of myeloma.

## Case presentation

A 68-year-old patient with no pathological history was followed for breast cancer, diagnosed after discovery by self-examination of a nodule in the left breast. The nodule was classified as ACR5 on mammographic ultrasound. The patient underwent a lumpectomy with axillary lymph node dissection. It was an infiltrating ductal carcinoma SBR III, hormone receptor positive, and Her2 negative. The tumor was classified as stage IIA. The patient received adjuvant chemotherapy (three cycles of anthracycline at a dose of 60 mg/m^2^ and cyclophosphamide at a dose of 600 mg/m^2^, followed by three cycles of docetaxel at a dose of 100 mg/m^2^), followed by radiotherapy and endocrine therapy (anastrozole 1 mg/d).

**Figure 1:**
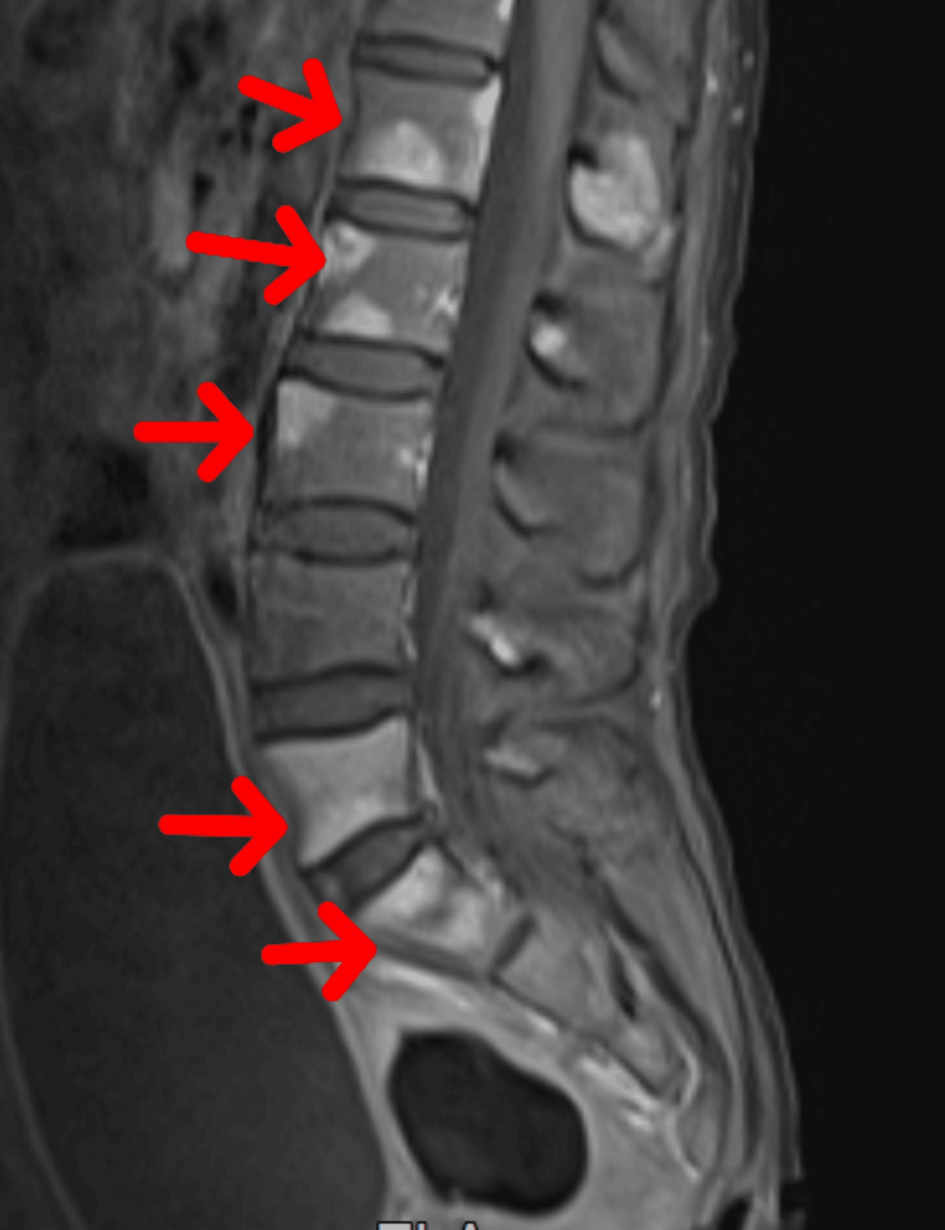
The spinal MRI: multiple vertebral lesions with hyperintensity on fat-suppressed T2-weighted imaging (arrows)

After two years, the evolution was marked by the appearance of inflammatory spinal pain, which prompted her to seek consultation. The physical examination found a patient with a performance status of 1, without tumor syndrome or signs of local relapse. The spinal MRI showed the presence of multiple vertebral lesions with hyperintensity on fat-suppressed T2-weighted imaging, mainly suggesting a relapse of her disease (Figure 1).

The initial biological exploration revealed a normal CA 15-3 level (7.3 U/mL) with isolated anemia (9 g/dL) on the complete blood count. The biochemical test showed hypoalbuminemia (32 g/L), moderate hypercalcemia (2.70 mmol/L), and elevated serum creatinine (160 µmol/L), indicating a glomerular filtration rate of 40 mL/min. Nevertheless, protein (89 g/L) and lactate dehydrogenase (200 U/L) levels were normal.

**Figure 2:**
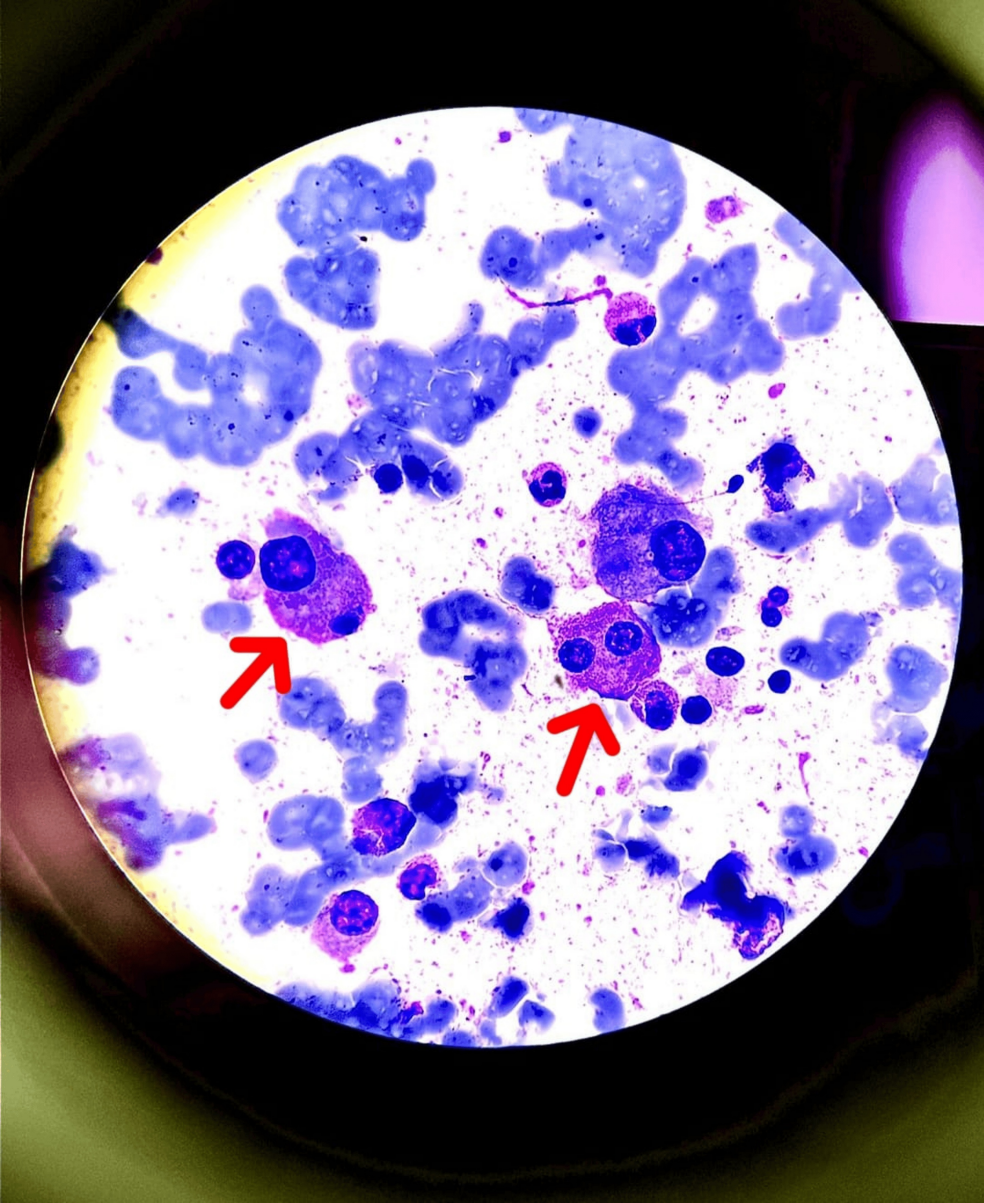
Bone marrow infiltration at 42% by predominantly dystrophic plasma cells (characterized by flaming cytoplasm, large size, and double nuclei)

In light of this clinical and biological presentation, a complementary biological assessment was necessary, notably protein electrophoresis, which showed a monoclonal hypergammaglobulinemic peak (43 g/L), and urine immunofixation electrophoresis, which demonstrated the presence of Bence-Jones protein. To confirm the diagnosis of myeloma, we performed a bone marrow examination that showed 42% infiltration of the bone marrow by dystrophic plasma cells (Figure 2).

In the end, we ruled out the diagnosis of breast cancer recurrence and retained the diagnosis of stage II multiple myeloma. The patient underwent six cycles of chemotherapy according to the VTD protocol (bortezomib 1.3 mg/m^2^ weekly, thalidomide 50 mg once a day, and dexamethasone 20 mg), marked by good tolerance. After six months of follow-up, the patient is in clinical and biological remission from her myeloma.

## Discussion

Breast cancer is the most common cancer among women, accounting for approximately 30% of newly diagnosed cancers [3]. It is the second leading cause of cancer death in women [3]. Screening and the development of adjuvant therapies have improved overall patient survival [4]. The risk of metastatic recurrence of early breast cancer is not negligible and is estimated to be between 20% and 30% [5].

However, the appearance of a bone lesion in a treated patient is not always related to a metastatic relapse of the already known cancer [6]. The incidence of malignant hemopathy, particularly multiple myeloma, in patients treated for breast cancer appears to be higher than in the general population [7]. Confirmation of bone relapse from breast cancer requires careful consideration. Several factors must be taken into consideration before starting treatment, including the patient's age, biological assessment, radiological appearance of the bone lesions, presence of other metastatic sites, and tumor markers [8].

The association of cytopenia, hypercalcemia, or lytic bone lesions in a patient pretreated for breast cancer can lead to confusion between two diagnoses: metastatic bone relapse and multiple myeloma [9]. Even bone scintigraphy cannot always distinguish between the two, as it is more sensitive to osteoblastic lesions but remains less reliable for lytic lesions [10,11]. Moreover, the presence of a bone abnormality on scintigraphy may be related to benign inflammatory or traumatic processes [9]. Fluorodeoxyglucose (FDG)-PET/CT remains the most sensitive for detecting bone and visceral recurrences of breast cancer [12]. The biopsy and the pathology study of bone lesions remain hard to perform [13]. With the development of molecular biology, a liquid biopsy from a blood sample appears to play a role in the diagnosis of relapses [14].

In addition to the previously mentioned biological abnormalities, the deterioration of renal function, the presence of a monoclonal spike during serum protein electrophoresis, and the search for Bence-Jones protein will lead us to consider the diagnosis of multiple myeloma more closely. The presence of clonal bone marrow plasmacytosis ≥ 10% on bone marrow biopsy, combined with the CRAB criteria, confirms the diagnosis of multiple myeloma [9,15].

## Conclusions

Breast cancer screening programs have promoted the diagnosis of this cancer in young populations and at an early stage, which has improved overall survival. The risk of developing other pathologies, particularly malignant hematological diseases in pretreated patients, does not seem negligible. Hence, it is important to ensure and conduct an etiological investigation before confirming a diagnosis of relapse and starting a treatment that may be harmful.

## References

[1] Registre des cancers de la région du Grand Casablanca 2008-2012. https://www.contrelecancer.ma/fr/documents/registre-des-cancers-de-la-region-du-grand-casab-3/.

[2] Coleman RE, Croucher PI, Padhani AR (2020). Bone metastases. Nat Rev Dis Primers.

[3] Siegel RL, Miller KD, Jemal A (2019). Cancer statistics, 2019. CA Cancer J Clin.

[4] Sancho-Garnier H, Colonna M (2019). Breast cancer epidemiology (Article in French). Presse Med.

[5] Kennecke H, Yerushalmi R, Woods R (2010). Metastatic behavior of breast cancer subtypes. J Clin Oncol.

[6] Zhang L, Wang Y, Gu Y, Hou Y, Chen Z (2019). The need for bone biopsies in the diagnosis of new bone lesions in patients with a known primary malignancy: a comparative review of 117 biopsy cases. J Bone Oncol.

[7] Jabagi MJ, Vey N, Goncalves A, Le Tri T, Zureik M, Dray-Spira R (2019). Evaluation of the incidence of hematologic malignant neoplasms among breast cancer survivors in France. JAMA Netw Open.

[8] Hough B, Brufsky A, Lentzsch S (2010). Metastatic breast cancer or multiple myeloma? Camouflage by lytic lesions. J Oncol.

[9] Edahiro T, Ureshino H, Yoshida T, Fukushima N, Ichinohe T (2023). Challenging diagnosis of lytic bone lesions between multiple myeloma and bone metastasis of primary breast cancer. Cureus.

[10] Liu T, Cheng T, Xu W, Yan WL, Liu J, Yang HL (2011). A meta-analysis of 18FDG-PET, MRI and bone scintigraphy for diagnosis of bone metastases in patients with breast cancer. Skeletal Radiol.

[11] Pires AO, Borges US, Lopes-Costa PV, Gebrim LH, da Silva BB (2014). Evaluation of bone metastases from breast cancer by bone scintigraphy and positron emission tomography/computed tomography imaging. Eur J Obstet Gynecol Reprod Biol.

[12] Colombié M, Goulon D, Kraeber-Bodéré F, Rousseau C (2014). Role of positron emission tomography (PET) in therapeutic assessment and follow-up of breast cancer. Médecine Nucléaire.

[13] Criscitiello C, André F, Thompson AM (2014). Biopsy confirmation of metastatic sites in breast cancer patients: clinical impact and future perspectives. Breast Cancer Res.

[14] Debré P, Ardaillou R, Galibert F. (2020). Liquid biopsy: alternative or complement to tissue biopsy?. Bulletin de l'Académie Nationale de Médecine.

[15] Rajkumar SV, Dimopoulos M A, Palumbo A (2014). International Myeloma Working Group updated criteria for the diagnosis of multiple myeloma. Lancet Oncology.

